# Association of familial history of diabetes or myocardial infarction and stroke with risk of cardiovascular diseases in four German cohorts

**DOI:** 10.1038/s41598-020-72361-4

**Published:** 2020-09-21

**Authors:** Kristin Mühlenbruch, Juliane Menzel, Marcus Dörr, Till Ittermann, Christa Meisinger, Annette Peters, Alexander Kluttig, Daniel Medenwald, Manuela Bergmann, Heiner Boeing, Matthias B. Schulze, Cornelia Weikert

**Affiliations:** 1 Department of Molecular Epidemiology, German Institute of Human Nutrition Potsdam-Rehbruecke, Nuthetal, Germany; 2 German Center for Diabetes Research (DZD), Neuherberg, Germany; 3 Department of Food Safety, German Federal Institute for Risk Assessment, Max-Dohrn-Str. 8-10, Berlin, Germany; 4 Department of Internal Medicine B, University Medicine Greifswald, Greifswald, Germany; 5 German Centre for Cardiovascular Research (DZHK), Partner Site Greifswald, Greifswald, Germany; 6 Independent Research Group Clinical Epidemiology, Helmholtz Zentrum München, German Research Center for Environmental Health, Neuherberg, Germany; 7 Chair of Epidemiology, Ludwig-Maximilians-Universität München, UNIKA-T Augsburg, Augsburg, Germany; 8 Institute of Epidemiology, Helmholtz Zentrum München, German Research Center for Environmental Health, Neuherberg, Germany; 9 Chair of Epidemiology, Ludwig-Maximilians-Universität München, Munich, Germany; 10 German Centre for Cardiovascular Research (DZHK), Partner Site Munich, Neuherberg, Germany; 11 Institute of Medical Epidemiology, Biostatistics and Informatics, Interdisciplinary Center for Health Sciences, Martin-Luther-University Halle-Wittenberg, Halle (Saale), Germany; 12 Department of Radiation Oncology, Martin-Luther-University Halle-Wittenberg, Halle (Saale), Germany; 13 Department of Epidemiology, German Institute of Human Nutrition Potsdam-Rehbruecke, Nuthetal, Germany; 14 Institute of Nutritional Sciences, University of Potsdam, Nuthetal, Germany

**Keywords:** Epidemiology, Risk factors

## Abstract

Since family history of diabetes is a very strong risk factor for type 2 diabetes, which is one of the most important risk factors for cardiovascular disease (CVD), it might be also useful to assess the risk for CVD. Therefore, we aimed to investigate the relationship between a familial (parents and siblings) history of diabetes and the risk of incident CVD. Data from four prospective German cohort studies were used: EPIC-Potsdam study (n = 26,054), CARLA study (n = 1,079), SHIP study (n = 3,974), and KORA study (n = 15,777). A multivariable-adjusted Cox regression was performed to estimate associations between familial histories of diabetes, myocardial infarction or stroke and the risk of CVD in each cohort; combined hazard ratios (HR_Meta_) were derived by conducting a meta-analysis. The history of diabetes in first-degree relatives was not related to the development of CVD (HR_Meta_ 0.99; 95% CI 0.88–1.10). Results were similar for the single outcomes myocardial infarction (MI) (HR_Meta_ 1.07; 95% CI 0.92–1.23) and stroke (HR_Meta_ 1.00; 95% CI 0.86–1.16). In contrast, parental history of MI and stroke were associated with an increased CVD risk. Our study indicates that diabetes in the family might not be a relevant risk factor for the incidence of CVD. However, the study confirmed the relationship between a parental history of MI or stroke and the onset of CVD.

## Introduction

Family history of diabetes is a well-known and strong risk factor for the onset of diabetes^1,2^ and was therefore included in many of the published diabetes prediction models^3,4^ . Interestingly, this is dependent on the number of affected relatives and the degree of relationship. A previous study observed that the number of affected parents and siblings might be more informative than general definitions of a family history^3^. Besides a direct link to the risk of developing diabetes, a few studies indicate that a family history of diabetes might also play a role in the development of cardiovascular diseases (CVD), suggested by the strong relationship between the prevalence of diabetes and risk of cardiovascular diseases^5^ as well as positive associations between family history of diabetes and cardiometabolic parameters. Accordingly, cross-sectional analyses observed that a family history of diabetes was associated with increased carotid artery intimal-medial thickness^6^ or endothelial dysfunction^7^. Moreover, parental history of diabetes was associated with higher levels of liver enzymes^8^, indicating an increased CVD risk^9,10^. In addition, the history of diabetes in male relatives was associated with a 1.4-fold (95% CI 1.1–1.7) higher risk for subclinical atherosclerosis^11^. As these findings were derived from cross-sectional studies and restricted to intermediate cardiometabolic markers, it remains controversial whether a family history of diabetes has any effect on the risk of future CVD. To date, only a very few prospective cohort studies have investigated the impact of family history of diabetes on the risk of future cardiovascular endpoints, with conflicting results^12–15^. Therefore, the present study hypothesized that diabetes in the family might be a relevant risk factor for the development of CVD in the future. Thus, the study aimed to investigate the associations between various patterns of a familial history of diabetes with the risk of incident CVD using data from four German cohort studies (EPIC-Potsdam study, CARLA study, SHIP study, KORA study). Accordingly, to confirm the general validity of the study findings, the present study also investigated the well-known association between familial histories of stroke and MI with incident CVD.

## Results

Table 1 shows the characteristics of the study participants from all four cohorts stratified by absence or presence of parental history of diabetes. Participants with a positive parental history of diabetes were more likely to be female and less educated. Moreover, participants with a positive parental history of diabetes were more likely to have prevalent hypertension, hyperlipidemia, or diabetes themselves and on average had a higher BMI and larger waist circumference. In addition, a positive family history of MI and stroke was also more likely in participants with a parental history of diabetes. In EPIC-Potsdam and CARLA, participants with a positive family history were slightly younger than participants with a negative family history, in contrast to SHIP and KORA. As depicted in Table 2, the distribution of a familial history of diabetes, myocardial infarction and stroke was very similar across all four cohorts.

**Table 1:** Characteristics of the study populations in german cohort studies by status of parental history of diabetes.

	EPIC-potsdam^b^		CARLA		SHIP		KORA	
	Parental history D− (n ~ 19,464^a^)	Parental history D+ (n ~ 6,590^a^)	Parental history D− (n = 758)	Parental history D+ (n = 321)	Parental history D− (n = 3,038)	Parental history D+ (n = 936)	Parental history D− (n = 9,230)	Parental history D+ (n = 2,551)
Incident CVD cases (%)	2.9	2.4	4.8	1.6	9.7	9.3	8.6	9.0
MI cases (%)	1.4	1.3	2.5	0.6	6.1	6.3	5.4	6.0
Stroke cases (%)	1.5	1.2	2.2	0.9	4.5	3.7	4.9	5.3
Sex (% male)	39.8	34.3	56.7	47.7	49.3	43.9	49.2	45.7
Age (years)	50.7 (9.00)	48.9 (8.66)	63.9 (0.4)	61.1 (0.5)	49.3 (17.0)	50.7 (13.5)	46.0 (13.8)	47.7 (11.9)
**Education (%)**								
No vocational training/vocational training	38.0	38.8	88.3	93.1	38.9^c^	38.6^c^		
Technical college	24.8	25.0	2.5	1.6	43.7^c^	48.3^c^		
University degree	37.2	36.2	9.2	5.3	17.5^c^	13.1^c^	34.4^d^	30.0^d^
**Smoking (%)**								
Never smoker	47.5	48.2	42.0	51.4	35.9	36.5	45.9	44.3
Former smoker	32.0	31.3	38.4	30.2	33.6	32.6	28.1	29.7
Current smoker < 20 cig./day	14.9	14.6	11.6	11.2	21.7	19.7	10.2	11.3
Current smoker ≥ 20 cig./day	5.52	5.9	8.1	7.2	8.8	11.2	15.9	14.8
**Sports activity (%)**								
≥ 2 h per week	22.0	22.8	17.9	18.7	44.2	38.5	19.9	17.5
**Anthropometry**								
BMI (kg/m^2^)	26.0 (4.19)	26.8 (4.69)	27.9 (0.16)	28.7 (0.28)	26.9 (4.73)	28.1 (4.83)	26.3 (4.3)	27.2 (4.5)
Waist circumference (cm)	85.7 (12.8)	87.0 (13.4)	99.0 (0.44)	100.0 (0.75)	88.4 (14.0)	90.9 (13.3)	88.0 (13.0)^e^	90.2 (13.1)^e^
Prevalent hypertension (%)	47.0	49.2	76.1	76.0	49.4	56.7	33.4	39.0
Prevalent hyperlipidemia (%)	27.2	28.0	37.9	40.8	20.1	26.8	28.0	32.2
Prevalent diabetes (%)	3.6	8.7	10.2	19.0	6.2	12.1	2.2	5.8
Family history of MI (% positive)	16.6	25.4	22.4	27.1	13.5	23.4	17.5^f^	29.6^f^
Family history of stroke (% positive)	18.7	28.6	24.3	37.1	16.5	23.2	19.1^f^	30.3^f^

Presented values are means and standard deviation for continuous variables and relative frequencies for categorical variables if not otherwise stated.

^a^Mean absolute frequencies across all imputations with varying numbers from one imputation dataset to the other.

^b^Means and standard errors after application of multiple imputation (m = 5).

^c^Education level obtained as < 10 years of school, = 10 years of school, > 10 years of school.

^d^≥ 12 years.

^e^For 11,926 persons only; ^f^ parental history of MI and stroke, respectively.

**Table 2:** Distribution of a familial history of diabetes, myocardial infarction, and stroke in EPIC-Potsdam, CARLA, SHIP, and KORA.

	EPIC-Potsdam (n ~ 26,054)	CARLA (n = 1,079)	SHIP (n = 3,974)	KORA (n = 11,781)
	% (n)	% (n)	% (n)	% (n)
**Diabetes**				
Parental history positive	25.3% (n ~ 6,590)	29.7% (n = 321)	23.6% (n = 936)	21.6% (n = 2,551)
Maternal history positive	17.5% (n ~ 4,562)	20.9% (n = 225)	16.3% (n = 646)	14.0% (n = 1,646)
Paternal history positive	10.3% (n ~ 2,687)	12.5% (n = 135)	9.0% (n = 359)	9.5% (n = 1,120)
Sibling history positive	5.7% (n ~ 1,473)	13.3% (n = 143)	7.0% (n = 272)	–
Family history positive	28.4% (n ~ 7,410)	36.8% (n = 397)	27.7% (n = 1,099)	–
**Myocardial infarction**				
Parental history positive	16.7% (n ~ 4,361)	19.9% (n = 215)	13.1% (n = 519)	20.1% (n = 2,369)
Maternal history positive	5.5% (n ~ 1,425)	7.0% (n = 75)	4.0% (n = 157)	5.9% (n = 697)
Paternal history positive	12.3% (n ~ 3,202)	14.5% (n = 156)	9.7% (n = 386)	15.4% (n = 1,818)
Sibling history positive	3.0% (n ~ 775)	6.1% (n = 66)	3.6% (n = 140)	–
Family history positive	18.8% (n ~ 4,903)	23.8% (n = 257)	15.8% (n = 629)	–
**Stroke**				
Parental history positive	19.4% (n ~ 5,055)	24.7% (n = 267)	16.3% (n = 647)	21.5% (n = 2,538)
Maternal history positive	11.5% (n ~ 2,986)	14.7% (n = 159)	8.6% (n = 341)	10.5% (n = 1,231)
Paternal history positive	9.1% (n ~ 2,374)	11.7% (n = 126)	8.3% (n = 331)	12.9% (n = 1,520)
Sibling history positive	2.4% (n ~ 624)	5.6% (n = 60)	3.0% (n = 116)	–
Family history positive	21.2% (n ~ 5,519)	28.1% (n = 301)	18.1% (n = 719)	–

### History of diabetes in the family and the risk of CVD

In all four cohort studies, the results from the Cox regression showed that a history of diabetes in any first-degree relative in the family was not significantly associated with a risk of developing CVD (Table 3). Consequently, the combined estimate from the meta-analysis was 0.99 (95% CI 0.88–1.10) for parental history of diabetes. As we adjusted for several anthropometric or lifestyle factors which might be included, and explain the relationship between diabetes in the family and incident CVD, unadjusted analyses (Supplemental Table S3) or adjustment for sex only (Supplemental Table S4) were also performed, which did not support relevant relationships between diabetes in the family and risk of CVD in the four cohorts. Of note, maternal history was associated with incident CVD in the crude and sex-adjusted model, but it is mainly driven by the KORA study (Supplemental Tables S3 and S4). Results were similar for the single outcomes myocardial infarction (MI) and stroke (Table 3) with HR_Meta_ 1.07 (95% CI 0.92–1.23) for MI and HR_Meta_ 1.00 (95% CI 0.86–1.16) for stroke. Furthermore, additional adjustment for prevalent diabetes did not affect the estimates for parental history of diabetes with CVD, MI and stroke.

**Table 3:** Hazard ratios for varying histories of diabetes in the family for incident cardiovascular diseases (CVD), stroke and myocardial infarction (MI).

Hazard Ratio^a^ (95% CI)	EPIC-potsdam (n ~ 26,054)	CARLA (n = 1,079)	SHIP (n = 3,974)	KORA (n = 11,781)	Pooled estimate ^b^
**Outcome: CVD**					
*Parental history of diabetes*	1.03 (0.83–1.29)	0.68 (0.37–1.24)	0.96 (0.75–1.24)	1.00 (0.86–1.17)	0.99 (0.88–1.10)
Maternal history	1.13 (0.91–1.40)	0.72 (0.37–1.42)	0.98 (0.75–1.28)	1.05 (0.89–1.24)	1.05 (0.93–1.18)
Paternal history	0.90 (0.64–1.27)	1.15 (0.52–2.53)	1.03 (0.68–1.55)	0.95 (0.75–1.21)	0.96 (0.81–1.14)
Sibling history	1.43 (0.94–2.17)	1.58 (0.90–2.78)	1.04 (0.73–1.47)	–	1.25 (0.97–1.61)
Family history	1.14 (0.92–1.40)	1.12 (0.69–1.82)	0.92 (0.73–1.16)	–	1.04 (0.90–1.21)
**Outcome: MI**					
*Parental history of diabetes*	1.17 (0.87–1.59)	0.55 (0.23–1.30)	1.07 (0.79–1.44)	1.06 (0.88–1.29)	1.07 (0.92–1.23)
Maternal history	1.26 (0.93–1.72)	0.53 (0.19–1.49)	1.13 (0.81–1.56)	1.09 (0.88–1.34)	1.12 (0.96–1.30)
Paternal history	1.08 (0.71–1.66)	0.82 (0.25–2.67)	0.85 (0.50–1.45)	1.00 (0.74–1.35)	0.99 (0.79–1.23)
Sibling history	**1.60 (1.01–2.54)**	1.24 (0.56–2.76)	1.28 (0.84–1.94)	–	**1.39 (1.04–1.86)**
Family history	**1.34 (1.02–1.75)**	0.83 (0.42–1.64)	1.03 (0.78–1.37)	–	1.14 (0.90–1.44)
**Outcome: Stroke**					
*Parental history of diabetes*	0.91 (0.68–1.22)	0.87 (0.38–2.03)	0.89 (0.60–1.32)	1.09 (0.90–1.33)	1.00 (0.86–1.16)
Maternal history	1.02 (0.76–1.37)	0.98 (0.40–2.39)	0.87 (0.57–1.33)	1.10 (0.88–1.37)	1.04 (0.88–1.22)
Paternal history	0.72 (0.41–1.26)	1.73 (0.58–5.11)	1.46 (0.79–2.69)	1.05 (0.77–1.44)	1.06 (0.79–1.43)
Sibling history	1.26 (0.48–3.28)	2.13 (0.95–4.74)	0.89 (0.54–1.49)	–	1.25 (0.73–2.14)
Family history	0.97 (0.70–1.35)	1.62 (0.80–3.29)	0.86 (0.60–1.21)	–	0.98 (0.76–1.27)

^a^Adjusted for sex, education, prevalent hypertension, BMI, waist circumference, smoking status, sports activity, alcohol intake and prevalent hyperlipidemia.

^b^ Pooled hazard ratios were derived from random effects model (REM).

Significant estimates with a 95% confidence interval greater than 1.0 are highlighted in bold.

With regard to homogeneity between the four different cohort studies, we observed a low variance between the studies, parental history of diabetes with tau-squared ranging between 0.0 and 0.0039 and similar HRs_Meta_. Although the CARLA study showed the largest difference from the respective cohorts, the low impact on the overall variance might be explained by the small sample size leading to a wider confidence interval.

### History of stroke or MI in the family and the risk of CVD

In contrast to diabetes in any part of the family, MI or stroke in the family was related to an increased CVD risk in all cohorts but CARLA. The HRs (95%-CI) for parental history of MI were 1.61 (1.32–1.95), 1.36 (1.01–1.83), 1.25 (1.08–1.46) and 1.23 (0.67–2.25) in EPIC-Potsdam, SHIP, KORA and CARLA, respectively. HR_Meta_ was 1.38 (95% CI 1.20–1.59) (Supplemental Table S1). A stronger association was observed for incident MI as a single outcome for all definitions of family history of MI; a parental history of MI showed an increased risk of HR_Meta_ 1.65 (95% CI 1.35–2.02). For incident stroke, the strongest associations were observed for a sibling history of MI with HR_Meta_ 1.52 (95% CI 1.02–2.26), and a family history of MI with HR_Meta_ 1.33 (95% CI 1.06–1.67) (Supplemental Table S1). Overall, strong associations between parental history of stroke and incident CVD, stroke and MI were observed (Supplemental Table S2). For sibling history or family history of stroke, this association showed a stronger positive trend than for the parent-related CVD or stroke risk. In the case of MI, a stronger risk was observed for parental history of stroke, maternal history of stroke and family history of stroke, with HR_Meta_ 1.22 (95% CI 1.00–1.49), 1.31 (95% CI 1.10–1.57) and 1.33 (95% CI 1.00–1.76), respectively (Supplemental Table S2).

### Mutual analyses of diabetes and CVD history

Cross-classifying family history of diabetes and CVD and mutual adjustment generally revealed a picture similar to the main analysis. We observed a positive association for those with a positive CVD history with CVD risk, either alone (HR_Meta_ 1.41, 95% CI 1.05–1.89) or in combination with a family history of diabetes (HR_Meta_ 1.37, 95% CI 0.91–2.08). Similarly, in models with mutual adjustment of family history of diabetes and CVD, only family history of CVD was related to an increased CVD risk (HR_Meta_ 1.39, 95% CI 1.03–1.88).

## Discussion

The results of the present analyses based on four population-based German cohorts do not support that diabetes in the family is a relevant risk factor for the development of cardiovascular diseases (MI and/or stroke). Specifically, we found no association between parental history of diabetes and incident CVD consistently across the cohort studies and in the meta-analysis. However, the present study confirmed the relationship between CVD (MI and/or stroke) in the family and the onset of CVD.

While there is scientific evidence for the relationship between diabetes in the family and the risk of developing diabetes^1,2^, we could not confirm the hypothesis that diabetes in the family might also be a relevant risk factor for the development of CVD. This is in contrast to previous studies which suggested a higher prevalence of CVD-related subclinical dysfunctions and risk factors such as increased carotid artery intimal-medial thickness, endothelial dysfunction, adverse cardiometabolic biomarker profile or subclinical atherosclerosis in persons with diabetes in the family^6–8,11^. However, these findings were derived from small intervention studies and were restricted to intermediate cardiometabolic markers; for a better comparison with our study findings, prospective cohort studies with incident CVD as the outcome need to be discussed. For example, in their study on developing combined risk prediction models for diabetes, CVD and chronic kidney disease (CKD), Alssema et al.^14^ reported a significant positive association between diabetes in the family and the combined outcome of diabetes, CVD and CKD. Additionally, they showed that diabetes in the family instead might play a stronger role in developing one or more of the respective diseases than MI or stroke in the family. This is in contrast to our findings, especially when comparing these results with our results for mutual adjustment of family history of diabetes and family history of CVD. With both types of family history information in the model, family history of CVD showed a strong increased and statistically significant risk for developing CVD, while a family history of diabetes did not. Mutual adjustment indicated that a family history of CVD might contain information that is also included in a family history of diabetes, but not vice versa. This might explain the stronger association between MI or stroke in the family and CVD, as the outcome as family history of diabetes might contain less relevant information than a family history of MI or stroke. However, this cannot be compared with the results by Alssema et al.^14^, since they only reported on the final complete model. The difference between the study results and the relevance of risk factors might be explained by the extended outcome with diabetes and CKD compared to only CVD in our study. Relevant risk factors might be more stronger related to the other outcomes—diabetes and CKD—rather than CVD, which is not distinguishable with a combined outcome. The results were generally conflicting among the few studies in this context^12,13,15^. On the one hand, it has been shown that diabetes in the family was a risk factor for coronary heart disease (OR 2.5, 95% CI 1.0–6.4) in women, but no such association was found in men (OR 0.4; 95% CI 0.2–1.1)^12^. On the other hand, Park et al. reported an increased risk for atherosclerotic cardiovascular diseases with a positive family history of diabetes (HR 1.10, 95% CI 1.05–1.15) in men, but no significant association among women (HR 0.98, 95% CI 0.92–1.05)^13^. However, these findings were based on sex-stratified analyses and are thus hard to compare with our results. Still, the importance of a family history of diabetes as a risk factor for cardiovascular disease remains controversial and information that is included in the construct of family history of diabetes is unknown. For incident diabetes, it was shown that with traditional anthropometric, lifestyle and genetic risk factors only 13% of the diabetes risk related to family history could be explained^2^, and for incident CVD it might be even less. In our study, we adjusted for several anthropometric or lifestyle factors which might be included and explain the relationship between diabetes in the family and incident CVD. However, crude association analyses did not support relevant relationships between diabetes in the family and risk of CVD in the four cohorts. Of note, maternal history was associated with incident CVD, but it is mainly driven by the KORA study (Supplemental Table S3). It could also be suggested that prevalent diabetes might be the strongest risk factor for the development of CVD and already contains the information of a family history; however, when adjusting the association of parental history of diabetes for it in our study, the results did not change.

In contrast to diabetes in the family, the well-known association between CVD (MI or stroke) in the family and the onset of CVD could be confirmed in the present study, which supports the general validity of our study findings. To date, numerous case–control studies have reported an approximately two- to fivefold higher prevalence of a positive family history among individuals with prevalent cardiometabolic diseases compared to controls^16–19^. Large prospective cohort studies also observed a positive association between self-reported parental or family history and future risk of cardiometabolic diseases, with multivariable-adjusted relative risks ranging from 0.8 to 2.2^20–23^. This is in line with our results.

While the present study included data from four German cohort studies, our investigation is the most comprehensive study on this topic. However, several limitations need to be considered. First, history of diabetes and CVD could obtain the chance of misclassification and uncontrolled confounding as the information on familial history based on self-reports. Furthermore, information on family history of diabetes and stroke in EPIC-Potsdam was obtained with the 5th follow-up and contained a lot of missing values. Therefore, for participants who had fatal CVD events before this follow-up, we did not have the information and might have constructed a more positive family history status by multiple imputation than it would be. However, by applying multiple imputation, we performed the most valid approach to handling missing data and confirmed our findings in three other German cohort studies. Still, studies showed differences in design, censoring, outcome definition and information on family history; however, standardized procedures were applied to achieve high quality data and all CVD cases were validated by medical records followed by an established protocol, and the observed associations did not show heterogeneity when combined with meta-analysis, which supports the general validity of our findings. Furthermore, we did not conduct sex-stratified analyses as reported in previous studies, however, this would affect the validity of our statistical models as the sample size would be small for the specific family history definitions and would rather be impossible for some cohorts.

In conclusion, our study does not support the hypothesis that diabetes in the family is a relevant risk factor for the development of cardiovascular diseases, including MI and stroke. However, the present study confirmed the relationship between a positive family history for CVD (MI or stroke) and the onset of CVD.

## Methods

### Study populations

Data from four different German cohort studies were used, the European Prospective Investigation into Cancer and Nutrition (EPIC)-Potsdam study, the Cardiovascular Disease, Living and Ageing in Halle (CARLA) study, the Study of Health in Pomerania (SHIP) and the Cooperative Health Research in the Region of Augsburg (KORA) study. All participants gave written informed consent. The studies followed the recommendations of the Declaration of Helsinki and were approved by the Ethics Committee of the Medical Association of the State of Brandenburg (EPIC-Potsdam), the Ethics Committee of the Medical Faculty of the University of Halle-Wittenberg (CARLA), University of Greifswald (SHIP), and by local authorities (KORA).

#### EPIC-potsdam study

The EPIC-Potsdam study is a prospective cohort study conducted among the general adult population of Potsdam, Germany, and surrounding municipalities. Overall, 27,548 participants, mainly within an age range of 35 to 65 years, were recruited between 1994 and 1998. Baseline assessment included physical examinations, a personal interview and questionnaires regarding lifestyle and nutrition^24^. Follow-up questionnaires were sent out every two to three years and were primarily used for determining the disease status of the participants^25^.

To identify potential CVD cases, self-reports, death certificates or linkage with hospital information systems were used. To increase sensitivity, the questionnaire included additional questions about typical stroke symptoms^26^. All identified potential CVD events were defined according to World Health Organization Monitoring of Trends and Determinants in Cardiovascular Disease criteria and classified by the International Statistical Classification of Diseases, 10th Revision (ICD-10). The details about the case identification and classification were described previously^26^. All verified incident cases of myocardial infarction (MI) and stroke up to September 2014 (5th follow-up round) were included. Information on diagnoses (diabetes, MI and stroke) occurring in the participants’ mother, father or siblings was obtained at the 5th follow-up round as well. After exclusion of participants with missing follow-up information (n = 589) or non-verified (n = 83) or prevalent (n = 822) cases of MI or stroke, 26,054 participants remained for the analysis. 359 MI cases and 363 stroke cases were observed within a mean follow-up time of 11 years. A multivariable multiple imputation was used to impute missing covariate information (age, sex, education, smoking status, physical activity, BMI, waist circumference, alcohol consumption, prevalent hypertension, prevalent hyperlipidemia) (n = 237) and missing family history information (n = 3,247). First, we included all variables of the analysis model in the imputation model and created a monotone pattern of missingness and second, we applied logistic regression with specifically defined models for all single family history variables in a next step.

#### CARLA study

The CARdiovascular Disease, Living and Ageing in Halle (CARLA) study is a prospective population-based cohort study of the elderly general population of the city of Halle in eastern Germany^27^. The CARLA cohort comprises 1,779 participants, aged between 45–83 years at baseline (967 men, 812 women). The baseline examination took place between December 2002 and January 2006. The first four-year follow-up examination was performed from March 2007 to March 2010 (mean follow-up time: 4.01 years). The net sample (after exclusion of deceased or non-responding people) then comprised 1,436 subjects, consisting of 790 men and 646 women aged between 50 and 87 years. In 2013, a second follow-up investigation was performed. The baseline and first follow-up examination consisted of a detailed medical examination (ECG, echocardiography, anthropometric measures, blood pressure measurements, taking of blood samples) and a standardized, computer-assisted interview. The second follow-up assessment comprised only a detailed computer-assisted interview and a blood pressure measurement. In addition, regular mortality follow-ups were carried out to get information on the vital status of the participants. In the case of a deceased person, the cause of death was defined as specified in the official death certificate compiled by the Federal Statistical Office.

The incidence of CVD was defined using self-reported Physician’s and physician (general practitioner) validated diagnoses of myocardial infarction or stroke between baseline and second follow-up. Type 2 diabetes was also defined using self-reported physician’s diagnoses of diabetes or intake of antidiabetic medication, coded according to the Anatomical Therapeutic Chemical Classification (ATC) system, where code A10 was selected to define the current use of antidiabetic medication. Information of a family history of diabetes, MI or stroke was obtained at the baseline examination.

After exclusion of participants with missing follow-up information (n = 227), non-verified CVD cases (n = 90), prevalent CVD at baseline (n = 94), missing covariate information (n = 24) or missing information regarding family histories of diabetes, MI or stroke (n = 265), 1,079 participants remained for the analysis. Within a mean follow-up time of 4.0 years, 21 MI cases and 20 stroke cases were observed.

#### SHIP study

The Study of Health in Pomerania (SHIP-0) is a cross-sectional population-based study conducted between 1997 and 2001 in West Pomerania, Germany^28^. The total population comprised 213,157 inhabitants^28^. A sample from the population aged 20 to 81 years was drawn from population registries^28^. A total of 7,008 persons were sampled with 292 persons of each sex in each of the 12 5-year age strata^28^. The net sample (without migrated or deceased persons) comprised 6,267 eligible participants^28^. Selected persons received a maximum of three written invitations^28^. In the case of non-response, letters were followed by a phone call or by home visits if contact by phone was not possible^28^. In the end, the SHIP population comprised 4,308 participants (corresponding to a final response of 68.8%)^28^, and due to missing information in covariates (education, smoking status, physical activity, BMI, waist circumference, prevalent hypertension, total cholesterol) (n = 120) or family histories (n = 214), 3,974 participants remained for analysis.

Information on vital status was collected at regular intervals starting from the time of enrolment. Death certificates were requested from the local health authority at the place of death and coded by a certified nosologist according to the International Classification of Diseases, 10th revision (ICD10)^28,29^. Two internists independently validated the underlying cause of death and performed a joint reading together with a third internist in cases of disagreement^28,29^.

Participants were censored at death or loss to follow-up. The number of months between baseline examination and censoring was used as the follow-up length^29^. The median duration of follow-up was 11.3 years. During the 224,884 person-years of follow-up, 382 participants died due to cardiovascular disease or survived a stroke or a myocardial infarction.

#### KORA Augsburg study

The prospective population-based KORA Augsburg study was conducted in the region of Augsburg (Augsburg city, counties Aichach-Friedberg and Augsburg), in Southern Germany. Between 1984/85 and 1999/2001, four independent cross-sectional surveys (S1–S4) were conducted among inhabitants of the study region aged between 25 and 74 years old (in the first survey, the participants were 25 to 64 years old)^30^.

Baseline information on sociodemographics, lifestyle, and medication intake was assessed, and a medical examination was performed. Family history for the respective diseases (diabetes, myocardial infarction and stroke) was defined as a parental history (yes vs. no).

All survey participants were followed up until 31 December 2009 to collect information on non-fatal stroke and MI cases via postal questionnaires. All self-reported incident cases of stroke and MI were validated through information from the treating physicians or the participants’ hospital records. In addition, regular mortality follow-ups were carried out for all survey participants. In the case of death during the follow-up, death certificates were requested from the local health authorities and the main cause of death was determined.

Altogether, 17,604 KORA participants were available. After exclusion of 1,060 persons due to missing follow-up information and 514 participants with prevalent MI or stroke, another 4,004 men and women has to be excluded due to missing values in the parental history and another 1819 persons due to missing covariate information (age, sex, education years, smoking status, physical activity, BMI, alcohol consumption, prevalent hypertension, prevalent hyperlipidemia). The data set for analysis included 11,781 study participants (5,708 men and 6,073 women). Within a mean follow-up time of 14.0 years, 652 MI cases and 592 stroke cases were observed.

### Patterns of a familial history of diabetes, stroke and MI

We defined a positive family history of diabetes if either the participant’s father, mother or at least one sibling had diabetes^3^. A positive parental history was defined as either the father or mother or both having diabetes^3^. Paternal history was positive if the father was diagnosed with diabetes, maternal history if the mother was diagnosed with diabetes, and sibling history if one or more siblings had a diabetes diagnosis^3^. The reference groups were always defined by the different definitions of familial history (family history, parental history, maternal history, sibling history), e.g. classifying individuals with maternal history; the reference group comprised those without a maternal history. The same definitions were applied for the different patterns of a familial history of stroke and MI. In KORA, information regarding a history of diabetes, stroke or MI in the family was only available for the father and mother, but not for the siblings.

### Statistical analysis

As outcome variables, we defined incident MI, incident stroke and incident cardiovascular disease (CVD) which contained either MI or stroke or both. For the analysis of a family history of diabetes, MI, or stroke with risk of MI, stroke or CVD, we applied Cox regression models adjusted for age, sex, education, body mass index (BMI), waist circumference, smoking behaviour, sports activity, alcohol consumption, prevalent hypertension and prevalent hyperlipidemia. Results from the Cox regression in the separate imputation datasets in EPIC-Potsdam were combined to an overall estimate by applying Rubin’s rules^31^.

Additionally, we investigated the association of solely CVD, solely diabetes or both diabetes and CVD in the family with risk of CVD, MI or stroke. To further investigate whether family history of diabetes and CVD include the same information, we mutually adjusted family history of diabetes and family history of CVD for each other.

The associations derived from single cohort studies were pooled by applying the meta-analysis (HR_Meta_); random-effects models were calculated according to DerSimonian and Laird^32^. As a measure for heterogeneity, we calculated tau-squared, which indicates the between-study variance.

As a sensitivity analysis, we additionally adjusted the association analysis between parental history of diabetes and CVD, MI and stroke for prevalent diabetes. Statistical analyses were performed with SAS (Versions 9.3 and 9.4, Enterprise Guide 6.1, SAS Institute Inc., Cary, NC, USA) or Stata 15.1 (Stata Corporation, College Station, TX, USA). For the computation of pooled estimates from the meta-analysis, we used PROC MIXED according to previously published methods^33,34^.

## Supplementary information

Supplementary Information.

## Data Availability

In accordance with German Federal and State data protection regulations, epidemiological data analyses of EPIC-Potsdam may be conducted upon application addressed to Prof. Dr. Heiner Boeing (boeing@dife.de). Each application has to pass a review process conducted by a scientific board. In KORA, for approved reasons, access restrictions apply to the data underlying the findings and thus they cannot be made freely available in the manuscript, the supplemental files, or a public repository. The data are subject to national data protection laws and restrictions were imposed by the ethics committee of the Bavarian Medical Association (“Bayerische Landesärztekammer”) to ensure data privacy of the study participants. However, access to the data can be applied for through an individual project agreement with KORA. Other interested authors can access the data in the same way the authors used to access the data. Applications for access to the data sets can be found at the following link: https://www.helmholtzmuenchen.de/en/kora-en/information-for-scientists/participating-in-kora/utilization-ofkora-data/index.html. With regard to the CARLA study, data access and blood sample use is possible in compliance with the rules and regulations of the CARLA steering committee. SHIP data are available upon request by means of a project agreement. Data can be applied for online (https://community-medicine.de) and access to it is subject to approval by the Steering Committee of the Research Network for Community Medicine.

## References

[1] Franks PW (2010). Diabetes family history: a metabolic storm you should not sit out. Diabetes.

[2] InterAct Consortium (2013). The link between family history and risk of type 2 diabetes is not explained by anthropometric, lifestyle or genetic risk factors: the EPIC-InterAct study. Diabetologia.

[3] Muhlenbruch K (2014). Update of the German Diabetes Risk Score and external validation in the German MONICA/KORA study. Diabetes Res. Clin. Pract..

[4] Buijsse B, Simmons RK, Griffin SJ, Schulze MB (2011). Risk assessment tools for identifying individuals at risk of developing type 2 diabetes. Epidemiol. Rev..

[5] Glovaci D, Fan W, Wong ND (2019). Epidemiology of diabetes mellitus and cardiovascular disease. Curr. Cardiol. Rep..

[6] Kao WH (2005). Family history of type 2 diabetes is associated with increased carotid artery intimal-medial thickness in Mexican Americans. Diabetes Care.

[7] Goldfine AB (2006). Family history of diabetes is a major determinant of endothelial function. J. Am. Coll. Cardiol..

[8] Abbasi A (2012). Parental history of type 2 diabetes and cardiometabolic biomarkers in offspring. Eur. J. Clin. Invest..

[9] Kunutsor SK, Apekey TA, Khan H (2014). Liver enzymes and risk of cardiovascular disease in the general population: a meta-analysis of prospective cohort studies. Atherosclerosis.

[10] Rahmani J (2019). Elevated liver enzymes and cardiovascular mortality: a systematic review and dose-response meta-analysis of more than one million participants. Eur. J. Gastroenterol. Hepatol..

[11] Scheuner MT, Setodji CM, Pankow JS, Blumenthal RS, Keeler E (2008). Relation of familial patterns of coronary heart disease, stroke, and diabetes to subclinical atherosclerosis: the multi-ethnic study of atherosclerosis. Genet. Med..

[12] Schumacher MC, Hunt SC, Williams RR (1990). Interactions between diabetes and family history of coronary heart disease and other risk factors for coronary heart disease among adults with diabetes in Utah. Epidemiology.

[13] Park JW (2008). Family history of diabetes and risk of atherosclerotic cardiovascular disease in Korean men and women. Atherosclerosis.

[14] Alssema M (2012). One risk assessment tool for cardiovascular disease, type 2 diabetes, and chronic kidney disease. Diabetes Care.

[15] Afarideh M (2017). Family history of diabetes and the risk of coronary heart disease in people with or without type 2 diabetes. Diabetes Metab.

[16] Shea S, Ottman R, Gabrieli C, Stein Z, Nichols A (1984). Family history as an independent risk factor for coronary artery disease. J. Am. Coll. Cardiol..

[17] Roncaglioni MC (1992). Role of family history in patients with myocardial infarction. An Italian case-control study. GISSI-EFRIM Investigators. Circulation.

[18] Pohjola-Sintonen S, Rissanen A, Liskola P, Luomanmaki K (1998). Family history as a risk factor of coronary heart disease in patients under 60 years of age. Eur. Heart J..

[19] Leander K, Hallqvist J, Reuterwall C, Ahlbom A, de Faire U (2001). Family history of coronary heart disease, a strong risk factor for myocardial infarction interacting with other cardiovascular risk factors: results from the Stockholm Heart Epidemiology Program (SHEEP). Epidemiology.

[20] Colditz GA (1986). A prospective study of parental history of myocardial infarction and coronary heart disease in women. Am. J. Epidemiol..

[21] Colditz GA (1991). A prospective study of parental history of myocardial infarction and coronary artery disease in men. Am. J. Cardiol..

[22] Sesso HD (2001). Maternal and paternal history of myocardial infarction and risk of cardiovascular disease in men and women. Circulation.

[23] Lloyd-Jones DM (2004). Parental cardiovascular disease as a risk factor for cardiovascular disease in middle-aged adults: a prospective study of parents and offspring. JAMA.

[24] Boeing H, Korfmann A, Bergmann MM (1999). Recruitment procedures of EPIC-Germany. European Investigation into Cancer and Nutrition. Ann. Nutr. Metab..

[25] Bergmann MM, Bussas U, Boeing H (1999). Follow-up procedures in EPIC-Germany-data quality aspects. European Prospective Investigation into Cancer and Nutrition. Ann. Nutr. Metab..

[26] Menzel J (2016). Omentin-1 and risk of myocardial infarction and stroke: Results from the EPIC-Potsdam cohort study. Atherosclerosis.

[27] Greiser KH (2005). Cardiovascular disease, risk factors and heart rate variability in the elderly general population: design and objectives of the CARdiovascular disease, Living and Ageing in Halle (CARLA) Study. BMC Cardiovasc. Disord..

[28] Volzke H (2011). Cohort profile: the study of health in Pomerania. Int. J. Epidemiol..

[29] Haring R (2010). Low serum testosterone levels are associated with increased risk of mortality in a population-based cohort of men aged 20–79. Eur. Heart J..

[30] Meisinger C (2002). Sex differences in risk factors for incident type 2 diabetes mellitus: the MONICA Augsburg cohort study. Arch Intern Med.

[31] Rubin DB (1987). Multiple Imputation for Nonresponse in Surveys.

[32] DerSimonian R, Laird N (1986). Meta-analysis in clinical trials. Control Clin. Trials.

[33] Witte, S. & Kuß, O. in *Proceedings der 5. Konferenz der SAS-Anwender in Forschung und Entwicklung (KSFE)* (ed E.; Streichfuss Schumacher, K.) 481–487 (Hohenheim, 2001).

[34] Kuss O, Koch A (1996). Meta-analysis macros for SAS. Comput. Stat. Data Anal..

