# Supplementary material for: Association of familial history of diabetes or myocardial infarction and stroke with risk of cardiovascular diseases in four German cohorts
Source: Sci Rep. 2020 Sep 21;10:15373. doi: 10.1038/s41598-020-72361-4 (PMC7505832; doi:10.1038/s41598-020-72361-4)
Supplement: Supplementary file 1 — Supplementary Information. [file 41598_2020_72361_MOESM1_ESM.docx]

**Association of familial history of diabetes or myocardial infarction and stroke with risk of cardiovascular diseases in four German cohorts**

Kristin Mühlenbruch^#^, Juliane Menzel^#^, Marcus Dörr, Till Ittermann, Christa Meisinger, Annette Peters, Alexander Kluttig, Daniel Medenwald, Manuela Bergmann, Heiner Boeing, Matthias B. Schulze, Cornelia Weikert

^#^ Shared first authorship

**Supplemental Table S1.** Hazard Ratios for varying histories of myocardial Infarction (MI) for incident cardiovascular diseases (CVD), MI and stroke

| Hazard Ratio ^a^ (95%-CI) | **EPIC-Potsdam**  (n~26054) | **CARLA**  (n=1079) | **SHIP**  (n=3974) | **KORA**  (n=11781) | **Pooled estimate ^b^** |
| --- | --- | --- | --- | --- | --- |
| **Outcome: CVD** |  |  |  |  |  |
| *Parental history of MI* | **1.61 (1.32-1.95)** | 1.23 (0.67-2.25) | **1.36 (1.01-1.83)** | **1.25 (1.08-1.46)** | **1.38 (1.20-1.59)** |
| Maternal history | **1.74 (1.25-2.42)** | 1.21 (0.44-3.37) | 1.42 (0.87-2.31) | 1.21 (0.95-1.53) | **1.38 (1.14-1.66)** |
| Paternal history | **1.52 (1.21-1.92)** | 1.42 (0.74-2.71) | 1.24 (0.88-1.75) | **1.25 (1.05-1.49)** | **1.33 (1.17-1.51)** |
| Sibling history | **2.76 (1.42-5.36)** | 1.51 (0.71-3.19) | 1.31 (0.88-1.97) | - | **1.68 (1.07-2.64)** |
| Family history | **1.88 (1.49-2.38)** | 1.41 (0.85-2.35) | **1.38 (1.07-1.79)** | - | **1.59 (1.27-1.99)** |
|  |  |  |  |  |  |
| **Outcome: MI** |  |  |  |  |  |
| *Parental history of MI* | **2.10 (1.61-2.72)** | 1.50 (0.72-3.13) | **1.46 (1.03-2.09)** | **1.49 (1.24-1.80)** | **1.65 (1.35-2.02)** |
| Maternal history | **2.35 (1.62-3.39)** | 1.69 (0.51-5.58) | 1.64 (0.95-2.88) | **1.49 (1.13-1.96)** | **1.76 (1.37-2.26)** |
| Paternal history | **1.85 (1.34-2.56)** | 1.78 (0.82-3.86) | 1.28 (0.84-1.95) | **1.43 (1.16-1.76)** | **1.51 (1.29-1.77)** |
| Sibling history | **4.13 (1.88-9.07)** | 1.61 (0.62-4.17) | 1.54 (0.92-2.57) | - | **2.12 (1.13-3.99)** |
| Family history | **2.61 (1.96-3.47)** | 1.60 (0.84-3.04) | **1.46 (1.07-2.00)** | - | **1.87 (1.22-2.88)** |
|  |  |  |  |  |  |
| **Outcome: Stroke** |  |  |  |  |  |
| *Parental history of MI* | 1.19 (0.85-1.67) | 0.88 (0.31-2.54) | 1.11 (0.68-1.79) | 0.93 (0.75-1.16) | 1.01 (0.85-1.20) |
| Maternal history | 1.27 (0.74-2.15) | 0.64 (0.09-4.82) | 1.02 (0.44-2.36) | 0.94 (0.67-1.31) | 1.02 (0.78-1.32) |
| Paternal history | 1.21 (0.86-1.72) | 0.93 (0.28-3.08) | 1.06 (0.61-1.87) | 0.95 (0.74-1.22) | 1.03 (0.85-1.25) |
| Sibling history | 1.67 (0.82-3.38) | 1.39 (0.41-4.69) | 1.47 (0.87-2.48) | - | **1.52 (1.02-2.26)** |
| Family history | 1.31 (0.97-1.77) | 1.16 (0.50-2.70) | 1.41 (0.97-2.05) | - | **1.33 (1.06-1.67)** |

^a^ Adjusted for sex, education, prevalent hypertension, BMI, waist circumference, smoking status, sports activity, alcohol intake and prevalent hyperlipidemia; ^b^ Pooled hazard ratios were derived from random effects model (REM)**Supplemental Table S2.** Hazard Ratios for varying histories of stroke for incident cardiovascular diseases (CVD), MI and stroke

| Hazard Ratio ^a^ (95%-CI) | **EPIC-Potsdam**  (n~26054) | **CARLA**  (n=1079) | **SHIP**  (n=3974) | **KORA**  (n=11781) | **Pooled estimate ^b^** |
| --- | --- | --- | --- | --- | --- |
| **Outcome: CVD** |  |  |  |  |  |
| *Parental history of stroke* | **1.30 (1.05-1.61)** | 1.12 (0.68-1.85) | 1.01 (0.79-1.30) | 1.15 (1.00-1.32) | **1.16 (1.04-1.28)** |
| Maternal history | 1.24 (0.95-1.62) | 1.25 (0.70-2.24) | 1.26 (0.94-1.71) | 1.18 (0.99-1.39) | **1.21 (1.07-1.37)** |
| Paternal history | **1.35 (1.04-1.76)** | 1.02 (0.51-2.05) | 0.82 (0.57-1.18) | 1.02 (0.86-1.21) | 1.06 (0.86-1.30) |
| Sibling history | 2.84 (0.86-9.42) | 0.54 (0.20-1.51) | **1.54 (1.01-2.33)** | - | 1.33 (0.62-2.85) |
| Family history | 1.57 (0.98-2.54) | 1.10 (0.68-1.78) | 1.11 (0.87-1.40) | - | 1.17 (0.97-1.42) |
|  |  |  |  |  |  |
| **Outcome: MI** |  |  |  |  |  |
| *Parental history of stroke* | **1.54 (1.18-1.99)** | 1.35 (0.72-2.52) | 1.00 (0.72-1.37) | 1.13 (0.96-1.35) | **1.22 (1.00-1.49)** |
| Maternal history | **1.49 (1.06-2.09)** | 2.10 (1.08-4.08) | 1.16 (0.79-1.72) | 1.21 (0.99-1.50) | **1.31 (1.10-1.57)** |
| Paternal history | **1.61 (1.12-2.30)** | 0.52 (0.16-1.69) | 0.94 (0.61-1.45) | 1.00 (0.81-1.24) | 1.09 (0.79-1.49) |
| Sibling history | 1.98 (0.87-4.53) | 0.24 (0.03-1.78) | 1.66 (0.98-2.83) | - | 1.44 (0.70-2.94) |
| Family history | **1.65 (1.22-2.23)** | 1.22 (0.66-2.27) | 1.11 (0.82-1.49) | - | **1.33 (1.00-1.76)** |
|  |  |  |  |  |  |
| **Outcome: Stroke** |  |  |  |  |  |
| *Parental history of stroke* | 1.11 (0.81-1.51) | 0.83 (0.36-1.92) | 1.16 (0.81-1.66) | **1.23 (1.03-1.47)** | **1.18 (1.03-1.38)** |
| Maternal history | 1.02 (0.67-1.56) | 0.36 (0.08-1.49) | 1.45 (0.95-2.20) | 1.21 (0.98-1.50) | 1.18 (0.93-1.49) |
| Paternal history | 1.13 (0.76-1.69) | 1.90 (0.78-4.64) | 0.77 (0.44-1.33) | 1.13 (0.92-1.40) | 1.11 (0.92-1.33) |
| Sibling history | 3.55 (0.51-24.7) | 0.98 (0.29-3.29) | 1.41 (0.78-2.55) | - | 1.41 (0.84-2.35) |
| Family history | 1.50 (0.59-3.86) | 0.93 (0.43-2.01) | 1.21 (0.85-1.71) | - | 1.19 (0.88-1.60) |

^a^ Adjusted for sex, education, prevalent hypertension, BMI, waist circumference, smoking status, sports activity, alcohol intake and prevalent hyperlipidemia; ^b^ Pooled hazard ratios were derived from random effects model (REM)

**Supplemental Table S3.** Hazard Ratios for Histories of Diabetes in the Family for Incident Cardiovascular Diseases (CVD)

| Hazard Ratio ^a^ (95%-CI) | **EPIC-Potsdam**  (n~26054) | **CARLA**  (n=1079) | **SHIP**  (n=3974) | **KORA**  (n=11781) | **Pooled estimate ^b^** |
| --- | --- | --- | --- | --- | --- |
| **Outcome: CVD** |  |  |  |  |  |
| *Parental history of stroke* | 1.05 (0.84-1.30) | 0.93 (0.40-2.14) | 0.95 (0.74-1.21) | 1.11 (0.95-1.28) | 1.06 (0.95-1.18) |
| Maternal history | 1.15 (0.93-1.43) | 1.10 (0.46-2.67) | 0.97 (0.74-1.27) | **1.33 (1.13-1.57)** | **1.18 (1.01-1.36)** |
| Paternal history | 0.89 (0.64-1.26) | 1.70 (0.59-4.87) | 0.99 (0.66-1.49) | 0.80 (0.62-0.99) | 0.86 (0.73-1.02) |
| Sibling history | 1.38 (0.90-2.11) | 2.00 (0.93-4.28) | 1.08 (0.77-1.51) | - | 1.28 (0.96-1.69) |
| Family history | 1.14 (0.92-1.40) | 1.61 (0.81-3.21) | 0.92 (0.73-1.15) | - | 1.07 (0.86-1.33) |

^a^ Unadjusted; ^b^ Pooled hazard ratios were derived from random effects model (REM)

**Supplemental Table S4.** Hazard Ratios for Histories of Diabetes in the Family for Incident Cardiovascular Diseases (CVD)

| Hazard Ratio ^a^ (95%-CI) | **EPIC-Potsdam**  (n~26054) | **CARLA**  (n=1079) | **SHIP**  (n=3974) | **KORA**  (n=11781) | **Pooled estimate ^b^** |
| --- | --- | --- | --- | --- | --- |
| **Outcome: CVD** |  |  |  |  |  |
| *Parental history of stroke* | 1.10 (0.88-1.36) | 1.00 (0.43-2.31) | 0.97 (0.76-1.25) | 1.08 (0.93-1.25) | 1.06 (0.95-1.18) |
| Maternal history | 1.21 (0.98-1.51) | 1.19 (0.49-2.88) | 1.00 (0.77-1.31) | **1.30 (1.11-1.53)** | **1.21 (1.08-1.36)** |
| Paternal history | 0.92 (0.66-1.30) | 1.76 (0.61-5.04) | 1.02 (0.68-1.54) | 0.76 (0.60-0.96) | 0.88 (0.72-1.08) |
| Sibling history | 1.49 (0.98-2.28) | 2.40 (1.10-5.21) | 1.13 (0.80-1.59) | - | 1.42 (0.99-2.02) |
| Family history | 1.20 (0.97-1.49) | 1.84 (0.92-3.70) | 0.95 (0.76-1.20) | - | 1.14 (0.88-1.47) |

^a^ Adjusted for sex; ^b^ Pooled hazard ratios were derived from random effects model (REM)
